# Data Driven Model-Free Adaptive Control Method for Quadrotor Formation Trajectory Tracking Based on RISE and ISMC Algorithm

**DOI:** 10.3390/s21041289

**Published:** 2021-02-11

**Authors:** Dongdong Yuan, Yankai Wang

**Affiliations:** School of Aerospace Engineering, Beijing Institute of Technology, Beijing 100081, China; 3120180112@bit.edu.cn

**Keywords:** quadrotor formation, formation cooperative trajectory tracking control, robust integral of the signum of the error, improved sliding mode control, data-driven model-free adaptive control

## Abstract

In order to solve the problems of complex dynamic modeling and parameters identification of quadrotor formation cooperative trajectory tracking control, this paper proposes a data-driven model-free adaptive control method for quadrotor formation based on robust integral of the signum of the error (RISE) and improved sliding mode control (ISMC). The leader-follower strategy is adopted, and the leader realizes trajectory tracking control. A novel asymptotic tracking data-driven controller of quadrotor is used to control the system using the RISE method. It is divided into two parts: The inner loop is for attitude control and the outer loop for position control. Both use the RISE method in the loop to eliminate interference and this method only uses the input and output data of the unmanned aerial vehicle(UAV) system and does not rely on any dynamics and kinematics model of the UAV. The followers realize formation cooperative control, introducing adaptive update law and saturation function to improve sliding mode control (SMC), and it eliminates the general SMC algorithm controller design dependence on the mathematical model of the UAV and has the chattering problem. Then, the stability of the system is proved by the Lyapunov method, and the effectiveness of the algorithm and the feasibility of the scheme are verified by numerical simulation. The experimental results show that the designed data-driven model-free adaptive control method for the quadrotor formation is effective and can effectively realize the coordinated formation trajectory tracking control of the quadrotor. At the same time, the design of the controller does not depend on the UAV kinematics and dynamics model, and it has high control accuracy, stability, and robustness.

## 1. Introduction

With the development and progress of artificial intelligence, avionics, inertial navigation system, and control technology, coupled with the characteristics of low cost, flexible operation, high stability, and strong adaptability of the micro rotorcraft. References [1,2] use unmanned aerial vehicle(UAV) in the military and civilian fields, but a single UAV has problems such as insufficient load, limited resources, and low efficiency in the face of complex conditions. It is difficult to ensure the smooth execution of diversified tasks in complex environments. The most reliable and effective solution to the above problems is to use multiple UAVs to form a coordinated formation [3], and it is used for fixed-wing aircraft. Compared to fixed-wing aircraft, the quadrotor has the advantage of vertical take-off and landing, hovering, and maneuvering flying, so it occupies a very important position in UAV systems [4,5]. Therefore, this paper uses multiple quadrotors to achieve formation control.

The coordinated formation control of multiple UAVs is an important technology in the autonomous collaborative control method of multiple UAVs. Multiple UAVs can be used to complete some complex tasks. Formation control strategies mainly include leader-follower method [6,7], behavior-based method [8], virtual structure method [9,10], consistency-based method [11], and so on. Among them, the leader-follower method control system is simple and easy to implement. For specific algorithms, reference [12] uses the backstepping control algorithm to realize UAV formation control well, but it depends on the accuracy of the mathematical model. References [13,14] use the intelligent learning control method to avoid this problem, but it has a large amount of calculation and poor anti-interference ability. Compared to the above method, References [15,16] using sliding mode control (SMC) can make the UAV system sliding on the sliding surface, which can reduce the system’s dependence on the accuracy of the mathematical model and enhance the system’s anti-interference ability. Therefore, it is widely used. However, its controller design still depends on the system model and the effect of SMC is not ideal under large disturbances. In addition, the sign function introduced in the design of SMC will cause chattering when the system tracks the target signal. Therefore, this paper introduces adaptive update law and saturation function to improve SMC, using the leader-follower method to realize formation control.

Trajectory tracking control is the basis for UAVs to achieve coordinated formation control. However, quadrotor UAVs are a complex system with strong coupling, strong nonlinearity, underdrive, and time-varying [17]. In addition, environmental disturbances, quadrotor under conditions such as abrasion and changes in payload, it is difficult to obtain or establish an accurate mathematical model of the UAV. Traditional model-based control methods are difficult to achieve effective control of the system. For quadrotor trajectory tracking control, reference [18,19] proposed a backstepping control method, but it has no processing mechanism for model uncertainty and lacks the ability to suppress external interference. References [20,21] proposed a linear quadratic control method, but it has poor portability and requires high precision for each parameter of the quadrotor. References [22,23] proposed an adaptive control method, but it is lacking effective processing capabilities for large external disturbances, such as wind disturbances. Reference [24] proposed a neural network control method, it has a large amount of calculation and cannot meet the requirements of real-time control of quadrotors. Therefore, this paper proposes the robust integral of the signum of the error (RISE) method to eliminate interference and this method only uses the input and output data of the UAV system, and does not rely on any UAV dynamics kinematics model.

Data-driven control, referring to the controller design, does not explicit or implicit contain the mathematical model information of the control process, and only uses the online or offline I/O data of the control system. It designs the controller through data processing and knowledge obtained, and it has convergence, stability, and robustness properties under certain assumptions, such as proportion integral derivative(PID) control [25], model-free adaptive control [26], and virtual reference feedback tuning control [27], etc. Current data-driven control technology is widely used in machinery manufacturing [28], motor control [29], transportation [30], and chemical production control [31], etc. In the field of unmanned aerial vehicles control, it is still less; reference [32] proposed a novel robust model-free adaptive control (Ro-MFAC) algorithm for quadrotor control with a class of unknown multiple-input multiple-output (MIMO) systems with measurement noise, but it is only for quadrotor attitude control. Reference [33] designed an iterative learning method, using a double-layer formation control system, and applied it to UAV formation, but it has a large amount of calculation and cannot meet the requirements of real-time performance.

Therefore, this paper adopts the inner and outer loop control strategy for the trajectory tracking control of the leader, and establishes the connection between inner and outer loop by designing the inverter and differentiator. The outer loop position control provides the required reference attitude angle for the inner loop, and the inner loop attitude control ensures the rapid convergence of the attitude angle. Both the inner and outer loop control adopt the RISE algorithm. The design of the controller does not depend on the mathematical model of the UAV and can compensate for external interference and modeling uncertainty. Aiming at the formation control of the leader and follower, this paper introduces an adaptive update law and saturation function to improve the SMC. The improved algorithm does not rely on the mathematical model of the system at all, and solves the chattering problem of the SMC. Using the above algorithm, this paper successfully realized the data-driven model-free adaptive trajectory tracking control of the quadrotor formation. The main contributions are as follows: (1) For the UAV trajectory tracking control, this paper designs the control system structure, adding the inverter and differentiator, using the inner and outer loops to use the RISE algorithm, introducing saturation function tracks the desired trajectory of the quadrotor to achieve trajectory tracking control, which can compensate for external interference and modeling uncertainty; (2) For the coordinated control of the leader and follower formations, leader performs the trajectory tracking control and the followers obtain the desired position according to the information of the leader after trajectory tracking. This paper also introduces an adaptive updated law and saturation function. The SMC eliminates the dependence of SMC on the system model and the chattering problem of SMC; (3) The trajectory tracking control and formation cooperative control algorithms in this paper only use the I/O data of the UAV and the UAV formation without any model information of UAVs and UAV formations, so it is a completely model-free method for coordinated trajectory tracking control of quadrotor formations. This paper also proves the stability of the system by the Lyapunov method, and verifies the effectiveness of the algorithm and the feasibility of the scheme by numerical simulation.

## 2. Data-Driven Model-Free Adaptive Trajectory Tracking Control Method of Leader Based on Rise

First of all, this chapter introduces the structure of the quadrotor control system. Secondly, in order to facilitate the understanding of the method proposed in the article and the need for simulation, this chapter introduces the kinematics and dynamics model of the quadrotor. Finally, you need to pay attention to the point that this chapter does not need the model information of the UAV when designing the data-driven model-free adaptive trajectory tracking control method of the leader.

### 2.1. Control System Structure and Mathematical Model

**Figure 1:**
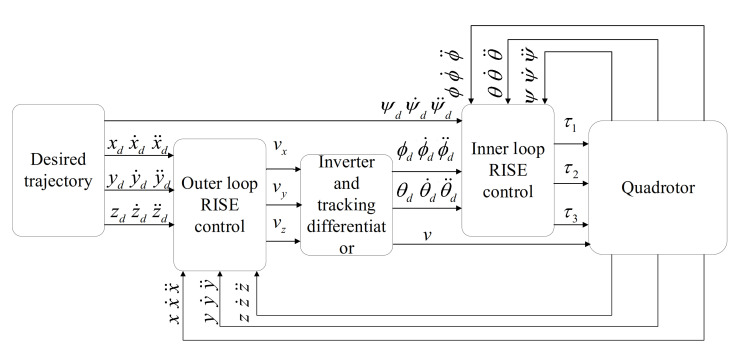
Leader trajectory tracking control system structure.

The design of the leader trajectory tracking control system mainly includes two closed loops. The outer loop position control is designed according to the position tracking error of the quadrotor, and the inverter and tracking differentiator provide the required reference attitude Euler angle for the inner loop attitude control. The inner loop attitude control is designed according to the error of the outer loop input reference attitude Euler angle and the system output Euler angle. The inner and outer loops all use the RISE method to finally realize the trajectory tracking control of the leader. The control system structure block diagram is shown in Figure 1.

**Figure 2:**
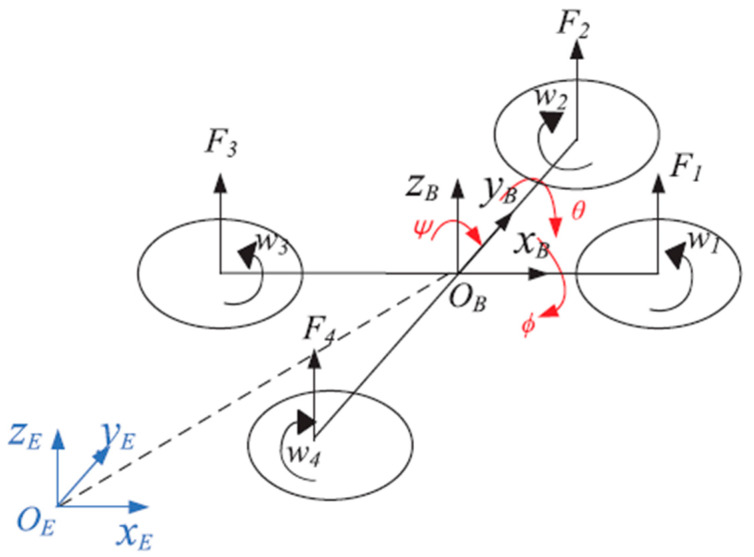
Schematic diagram of quadrotor.

The schematic diagram of the quadrotor is shown in Figure 2. It has six degrees of freedom but only four actuators. It is a multi-input, multi-output, strong coupling, and under-drive system. Define $x_{E}y_{E}z_{E}o_{E}$ as the world coordinate system, $x_{B}y_{B}z_{B}o_{B}$ as the body coordinate system, x, y, z are three position coordinates respectively, θ, ϕ, ψ are three Euler angles respectively, the rotation matrix is show as Equations (1)–(3):

(1) RBE(ϕ)=1000cosϕ−sinϕ0sinϕcosϕ,

(2) RBE(θ)=cosθ0sinθ010−sinθ0cosθ,

(3) RBE(ψ)=cosψ−sinψ0sinψcosψ0001.

Therefore, the coordinate conversion matrix from the linear and angular velocity body coordinate system of the quadrotor UAV to the world coordinate system is shown in Equations (4) and (5):

(4) RBE=cθcψsϕsθcψ−cϕsψcϕsθcψ+sϕsψcθsψsϕsθsψ+cϕcψcϕsθsψ−sϕcψ−sθsϕcθcϕcθ,

(5) RBEa=1sϕtθcϕtθ0cϕ−sϕ0sϕ/cθcϕ/cθ.

**Remark** **1.** *The attitude angles ϕ and θ are bounded as $\phi \in (-\frac{\pi }{2},\frac{\pi }{2})$ and $\theta \in (-\frac{\pi }{2},\frac{\pi }{2})$.*

Among them, c⋅, s⋅, t⋅ represent cos(), sin(), tan() respectively, and RBE, RBEa are the coordinate transformation matrix from the linear velocity and angular velocity body coordinate system to the world coordinate system.

Define ω_1_, ω_2_, ω_3_, ω_4_ to be the angular velocity of the four rotors, f_1_, f_2_, f_3_, and f_4_ are the thrusts of the four rotors respectively. The dynamics and kinematics model of the quadrotor are shown in Equations (6)–(9):

(6) $$f_{i}=K_{T}\omega_{i}{}^{2},i=1,2,3,4,$$

(7) $$\left[\begin{array}{c} u\\ \tau_{1}\\ \tau_{2}\\ \tau_{3} \end{array}\right]=\left[\begin{array}{cccc} 1 & 1 & 1 & 1\\ 0 & d & 0 & -d\\ -d & 0 & d & 0\\ -k_{c} & k_{c} & -k_{c} & k_{c} \end{array}\right]\left[\begin{array}{c} f_{1}\\ f_{2}\\ f_{3}\\ f_{4} \end{array}\right],$$

(8) $$m\;\ddot{\xi }=-K_{\xi }\;\dot{\xi }+v-mge+d_{\xi },$$

(9) $$J\ddot{\eta }=-K_{\eta }\dot{\eta }+\tau +d_{\eta }.$$

**Assumption** **1.** *The unknown time-varying disturbances $d_{\xi }$ and $d_{\eta }$ are bounded and the derivative of $d_{\eta }$ is also bounded.*

**Assumption** **2.** *The state variables ξ and η and their derivatives $\;\dot{\xi }$ and $\dot{\eta }$ are measurable.*

Where $K_{T}$ is the thrust coefficient depending on the characteristics of the rotor blade. u is the total thrust, $\tau_{1}$, $\tau_{2}$, $\tau_{3}$ are the roll, pitch, and yaw moments respectively, d is the distance from the rotor center to the center of mass, and $k_{c}$ is the force-torque ratio factor. m is the mass of the quadrotor, $\xi ={\left[\begin{array}{ccc} x & y & z \end{array}\right]}^{T}$ is the movement position in three directions, $K_{\xi }=diag(K_{\xi 1},K_{\xi 2},K_{\xi 3})$ is the position aerodynamic damping matrix, $v={\left[\begin{array}{ccc} v_{x} & v_{y} & v_{z} \end{array}\right]}^{T}$ is the virtual reference input, $e={\begin{array}{ccc} 0 & 0 & 1 \end{array}}^{T}$ is a constant matrix, $d_{\xi }={\left[\begin{array}{ccc} d_{\xi }{}_{1} & d_{\xi }{}_{2} & d_{\xi }{}_{3} \end{array}\right]}^{T}$ is the position disturbance. $J=diag(J_{x},J_{y},J_{z})$ is the moment of inertia matrix, $\eta ={\begin{array}{ccc} \phi & \theta & \psi \end{array}}^{T}$ is the three attitude Euler angles, $K_{\eta }=diag(K_{\eta 1},K_{\eta 2},K_{\eta 3})$ is the attitude rotation aerodynamic damping matrix, $\tau ={\left[\begin{array}{ccc} \tau_{1} & \tau_{2} & \tau_{3} \end{array}\right]}^{T}$ is the rotation input torque, and $d_{\eta }={\left[\begin{array}{ccc} d_{\eta }{}_{1} & d_{\eta }{}_{2} & d_{\eta }{}_{3} \end{array}\right]}^{T}$ is the unknown disturbance.

Considering Remark 1, according to [34,35], the design of virtual control input $v={\left[\begin{array}{ccc} v_{x} & v_{y} & v_{z} \end{array}\right]}^{T}$ is shown in Equation (10):

(10) $$\left\{\begin{array}{c} v_{x}=(\cos\psi \sin\theta \cos\phi +\sin\phi \sin\psi )u\\ v_{y}=(\sin\psi \sin\theta \cos\phi -\sin\phi \cos\psi )u\\ v_{z}=(\cos\theta \cos\phi )u \end{array}\right..$$

Define $\phi_{d}$, $\theta_{d}$ as the reference attitude Euler angles, then the design of inverter is as (11):

(11) $$\left\{\begin{array}{c} \phi_{d}=\sin^{-1}(\frac{v_{x}\sin\psi_{d}-v_{y}\cos\psi_{d}}{u})\\ \theta_{d}=\tan^{-1}(\frac{v_{x}\cos\psi_{d}+v_{y}\sin\psi_{d}}{v_{z}})\\ u=\sqrt{v_{x}{}^{2}+v_{y}{}^{2}+v_{z}{}^{2}} \end{array}\right.,$$

when u=0 is equal to 0, Equation (11) has singularities, This is a disadvantage of the algorithm proposed in this paper, at this time we have to avoid it by tuning parameters.

In addition, in order to obtain the values of ${\ddot{\phi }}_{d}(t)$, ${\dot{\phi }}_{d}(t)$, ${\ddot{\theta }}_{d}(t)$, ${\dot{\theta }}_{d}(t)$ in Figure 1, the tracking differentiator is designed in Equation (12):

(12) $$\left\{\begin{array}{c} {\dot{x}}_{1\phi }=x_{2\phi }\\ {\dot{x}}_{2\phi }=-r_{\phi }sign(x_{1\phi }-\phi (t)+\frac{x_{2\phi }\left|x_{2\phi }\right|}{2r_{\phi }}) \end{array}\right..$$

**Remark** **2.** *In order to avoid sudden changes in the motion of the quadrotor, the reference trajectory $\xi_{d}$, $\eta_{d}$ are designed to meet $\xi_{d}^{\left(i\right)}(t)\in L_{\infty },\eta_{d}^{\left(j\right)}(t)\in L_{\infty }$, where (i),(j) represent the i−th,j−th order differentials of time, i=1,2,j=1,2,3.*

Where ϕ(t) is the differential input signal, $x_{1\phi }$ tracks ϕ(t), $x_{2\phi }$ tracks $\dot{\phi }(t)$, ${\dot{x}}_{2\phi }$ tracks $\ddot{\phi }(t)$, and $r_{\phi }$ is a constant that determines the tracking speed. Bring $\phi (t)=\phi_{d}(t)$ or $\phi (t)=\theta_{d}(t)$ into Equation (12) to get ${\ddot{\phi }}_{d}(t)$, ${\dot{\phi }}_{d}(t)$, ${\ddot{\theta }}_{d}(t)$, ${\dot{\theta }}_{d}(t)$ as shown in Equations (13) and (14):

(13) $${\ddot{\phi }}_{d}(t)\approx -r_{\phi }sign(x_{1\phi }-\phi_{d}(t)+\frac{x_{2\phi }\left|x_{2\phi }\right|}{2r_{\phi }}),{\dot{\phi }}_{d}(t)\approx \int_{0}^{t}-r_{\phi }sign(x_{1\phi }-\phi_{d}(\tau )+\frac{x_{2\phi }\left|x_{2\phi }\right|}{2r_{\phi }})d\tau ,$$

(14) $${\ddot{\theta }}_{d}(t)\approx -r_{\phi }sign(x_{1\phi }-\theta_{d}(t)+\frac{x_{2\phi }\left|x_{2\phi }\right|}{2r_{\phi }}),{\dot{\theta }}_{d}(t)\approx \int_{0}^{t}-r_{\phi }sign(x_{1\phi }-\theta_{d}(\tau )+\frac{x_{2\phi }\left|x_{2\phi }\right|}{2r_{\phi }})d\tau .$$

**Remark** **3.** *Equations (13) and (14) make the ${\dot{\phi }}_{d}(t)$ and ${\dot{\theta }}_{d}(t)$ become continuous.*

### 2.2. Outer Loop Position Tracking Control

The description of the outer loop control system of the quadrotor is shown in Equation (8). Let the position tracking error $e_{\xi 1}$ and the filtered error signals $e_{\xi 2}$, $e_{\xi 3}$ be defined as follows [36,37]:

(15) $$e_{\xi 1}{=\xi }_{d}-\xi ,$$

(16) $$e_{\xi 2}={\dot{e}}_{\xi 1}+\lambda e_{\xi 1},$$

(17) $$e_{\xi 3}={\dot{e}}_{\xi 2}+\sigma e_{\xi 2},$$

where ${\dot{e}}_{\xi 2}={\ddot{e}}_{\xi 1}+\lambda {\dot{e}}_{\xi 1}$, ${\ddot{e}}_{\xi 1}={\ddot{\xi }}_{d}-\ddot{\xi }$ and ${\dot{e}}_{\xi 1}={\dot{\xi }}_{d}-\dot{\xi }$, which is determined by the error of input and output data of the system. $\lambda =diag\{\lambda_{1},\lambda_{2},\lambda_{3}\}$, $\sigma =diag\{\sigma_{1},\sigma_{2},\sigma_{3}\}$ are positive definite diagonal matrices, and $\lambda_{i}>\frac{1}{2},\sigma_{i}>\frac{1}{2},i=1,2,3$. In the text, the input data are $x_{d},{\dot{x}}_{d},{\ddot{x}}_{d},y_{d},{\dot{y}}_{d},{\ddot{y}}_{d},z_{d},{\dot{z}}_{d},{\ddot{z}}_{d}$ and output data are $x,\dot{x},\ddot{x},y,\dot{y},\ddot{y},z,\dot{z},\ddot{z},\phi ,\dot{\phi },\ddot{\phi },\theta ,\dot{\theta },\ddot{\theta },\psi ,\dot{\psi },\ddot{\psi }$.

Combine the Equations (15)–(17) and (8) obtain Equation (18):

(18) $$m{\dot{e}}_{\xi 3}=m\sigma {\dot{e}}_{\xi 2}+m\lambda {\ddot{e}}_{\xi 1}+m{\dddot{\xi }}_{d}+K_{\xi }\ddot{\xi }-\dot{v}-{\dot{d}}_{\xi }.$$

Define the auxiliary equations $N_{1}(t)$, $N_{d1}(t)$, ${\tilde{N}}_{1}(t)$ as Equations (19)–(21):

(19) $$N_{1}(t)=K_{\xi }\ddot{\xi }-{\dot{d}}_{\xi }+m{\dddot{\xi }}_{d}+m\lambda {\ddot{e}}_{\xi 1}+m\sigma {\dot{e}}_{\xi 2}+e_{\xi 2},$$

(20) $$N_{d1}(t)=K_{\xi }{\ddot{\xi }}_{d}-{\dot{d}}_{\xi }+m{\dddot{\xi }}_{d},$$

(21) $${\tilde{N}}_{1}(t)=N_{1}(t)-N_{d1}(t)=-K_{\xi }{\ddot{e}}_{\xi 1}+m\lambda {\ddot{e}}_{\xi 1}+m\sigma {\dot{e}}_{\xi 2}+e_{\xi 2}.$$

According to Equations (19)–(21), Equation (18) can be written as Equation (22):

(22) $$m{\dot{e}}_{\xi 3}=-e_{\xi 2}-\dot{v}+N_{d1}(t)+{\tilde{N}}_{1}(t).$$

According to Remark 2, we know that $N_{d1}(t),{\dot{N}}_{d1}(t)\in L_{\infty }$, and $N_{1}(t)$ is continuously differentiable. According to the mean-value theorem in [36], so ${\tilde{N}}_{1}(t)$ is bounded as in Equation (23):

(23) $$\left\|{\tilde{N}}_{1}(t)\right\|\leq \rho_{1}\left\|\Gamma_{1}\right\|,$$

where $\left\|\cdot \right\|$ represents the Euclidean norm, $\Gamma_{1}={\left[e_{\xi 1},e_{\xi 2},e_{\xi 3}\right]}^{T}$ is the error vector, and $\rho_{1}$ is a positive constant. According to references [37,38], the RISE position controller is designed as follows:

(24) $$v=\int_{0}^{t}(K_{s1}+I_{3\times 3})e_{\xi 3}d\tau +\int_{0}^{t}\beta_{1}sign(e_{\xi 2})d\tau .$$

Among them, $K_{s1}=diag(K_{s11},K_{s12},K_{s13})$, $\beta_{1}=diag(\beta_{11},\beta_{12},\beta_{13})$ are positive definite gain control matrices, and $I_{3\times 3}$ is an identity matrix.

**Remark** **4.** *Equation (24) contains a sign function sign(⋅), which has chattering problems. The saturation Equation (25) is introduced to replace the sign function sign(⋅). The final RISE controller design is as shown in Equation (26):*

(25) $$\tanh(x)=\frac{\sinh(x)}{\cosh(x)}=\frac{e^{x}-e^{-x}}{e^{x}+e^{-x}},$$

(26) $$v=\int_{0}^{t}(K_{s1}+I_{3\times 3})e_{\xi 3}d\tau +\int_{0}^{t}\beta_{1}\tanh(e_{\xi 2})d\tau .$$

**Remark** **5.** *Equation (26) is only related to the system error data $e_{\xi 1}$ and its filtered error signals $e_{\xi 2}$, $e_{\xi 3}$, but not rely on the system model. Therefore, the proposed outer loop position RISE algorithm is strictly a data-driven model-free control method.*

To prove the stability, define the auxiliary Equation as follows:

(27) $$Q(t)=e_{\xi 3}{}^{T}(N_{d1}(t)-\beta_{1}\tanh(e_{\xi 2})).$$

If the control matrix $\beta =diag(\beta_{1},\beta_{2},\beta_{3})$ satisfies:

(28) $$\beta_{1i}>{\left\|N_{d1}(t)\right\|}_{\infty }+\frac{1}{\sigma_{i}}{\left\|{\dot{N}}_{d1}(t)\right\|}_{\infty },$$

where ${\left\|\cdot \right\|}_{\infty }$ represents the infinite norm. Then:

(29) $$\int_{0}^{t}Q(\tau )d\tau \leq \varpi .$$

The positive constant ϖ is defined as:

(30) $$\varpi ={\left\|\beta_{1}e_{\xi 2}(0)\right\|}_{1}-e_{\xi 2}{}^{T}(0)N_{d1}(0).$$

Combine Equation (17) into Equation (27), we get:

(31) $$\begin{array}{cc} \int_{0}^{t}Q(\tau )d\tau = & \int_{0}^{t}e_{\xi 2}(\tau )\sigma (N_{d1}(\tau )-\beta_{1}\tanh(e_{\xi 2}(\tau )))d\tau +\\ & \int_{0}^{t}\frac{de_{\xi 2}{}^{T}(\tau )}{d\tau }N_{d1}(\tau )d\tau -\int_{0}^{t}\frac{de_{\xi 2}{}^{T}(\tau )}{d\tau }\beta_{1}\tanh(e_{\xi 2}))d\tau \\ = & \int_{0}^{t}e_{\xi 2}(\tau )\sigma (N_{d1}(\tau )-\beta_{1}\tanh(e_{\xi 2}(\tau )))d\tau +\\ & (e_{\xi 2}{}^{T}(\tau )N_{d1}(\tau ))\left|{}_{0}^{t}\right.-\int_{0}^{t}e_{\xi 2}{}^{T}\frac{d(N_{d1}(\tau ))}{d\tau }d\tau -\sum_{i=1}^{3}\beta_{1i}\left|e_{\xi 2i}(\tau )\right|\left|{}_{0}^{t}\right.\\ = & \int_{0}^{t}e_{\xi 2}{}^{T}(\tau )\sigma (N_{d1}(\tau )-\sigma^{-1}\frac{d(N_{d}(\tau ))}{d\tau }-\beta_{1}\tanh(e_{\xi 2}(\tau )))d\tau +\\ & e_{\xi 2}{}^{T}(t)N_{d1}(t)-e_{\xi 2}{}^{T}(0)N_{d1}(0)+\sum_{i=1}^{3}\beta_{1i}\left|e_{\xi 2i}(0)\right|-\sum_{i=1}^{3}\beta_{1i}\left|e_{\xi 2i}(t)\right| \end{array}$$

Then, an upper bound is obtained as follows:

(32) $$\begin{array}{cc} \int_{0}^{t}Q(\tau )d\tau \leq & \int_{0}^{t}\left|e_{\xi 2}{}^{T}(\tau )\sigma \right|(\left|N_{d1}(\tau )\right|+\sigma^{-1}\left|\frac{d(N_{d}(\tau ))}{d\tau }\right|-\beta_{1})d\tau +\\ & \sum_{i=1}^{3}\left|e_{\xi 2i}(t)\right|(N_{d1i}(t)-\beta_{1i})+\sum_{i=1}^{3}\beta_{1i}\left|e_{\xi 2i}(0)\right|-e_{\xi 2}{}^{T}(0)N_{d1}(0)\\ \leq & \sum_{i=1}^{3}\beta_{1i}\left|e_{\xi 2i}(0)\right|-e_{\xi 2}{}^{T}(0)N_{d1}(0) \end{array}$$

If $\beta_{1i}(i=1,2,3)$ is selected according to Equation (28). Then:

(33) $$\begin{array}{l} \int_{0}^{t}Q(\tau )d\tau \leq \sum_{i=1}^{3}\beta_{1i}\left|e_{\xi 2i}(0)\right|-e_{\xi 2}{}^{T}(0)N_{d1}(0)\\ \;={\left\|\beta_{1}e_{\xi 2}(0)\right\|}_{1}-e_{\xi 2}{}^{T}(0)N_{d1}(0) \end{array}$$

Therefore:

(34) $$\int_{0}^{t}Q(\tau )d\tau \leq \varpi .$$

Define the Lyapunov function as follows:

(35) $$V(\Gamma_{1},t)=\frac{1}{2}e_{\xi 1}{}^{T}e_{\xi 1}+\frac{1}{2}e_{\xi 2}{}^{T}e_{\xi 2}+\frac{1}{2}e_{\xi 3}{}^{T}me_{\xi 3}+\alpha (t),$$

where α(t) is defined as follows:

(36) $$\alpha (t)=\varpi -\int_{0}^{t}Q(\tau )d\tau .$$

It can be seen from Equation (34) that α(t)>0, therefore the Lyapunov function Equation (35) is positive definite, and the differential of $V(\Gamma_{1},t)$ with respect to time is as follows:

(37) $$\dot{V}(\Gamma_{1},t)=e_{\xi 1}{}^{T}{\dot{e}}_{\xi 1}+e_{\xi 2}{}^{T}{\dot{e}}_{\xi 2}+e_{\xi 3}{}^{T}m{\dot{e}}_{\xi 3}+\dot{\alpha }(t).$$

Putting Equation (19) into Equation (21) and Equation (26) into Equation (8) can obtain the closed-loop subsystem of $e_{\xi 3}$ as follows:

(38) $$m{\dot{e}}_{\xi 3}=-e_{\xi 2}-(K_{s1}+I_{3\times 3})e_{\xi 3}-\beta_{1}\tanh(e_{\xi 2})+{\tilde{N}}_{1}(t)+N_{d1}(t).$$

Putting Equation (15) into Equation (16), Equation (38) into Equation (37), we get:

(39) $$\begin{array}{ll} \dot{V}(\Gamma_{1},t)= & e_{\xi 1}{}^{T}e_{\xi 2}-e_{\xi 1}{}^{T}\lambda e_{\xi 1}-e_{\xi 2}{}^{T}\sigma e_{\xi 2}-e_{\xi 3}{}^{T}K_{s1}e_{\xi 3}-\\ & e_{\xi 3}{}^{T}e_{\xi 3}+e_{\xi 3}{}^{T}{\tilde{N}}_{1}(t)+[e_{\xi 3}{}^{T}(N_{d1}(t)-\beta_{1}\tanh(e_{\xi 2}))-Q(t)] \end{array}$$

Combine Equations (23), (27) and (39), and $e_{\xi 1}{}^{T}e_{\xi 2}\leq \frac{1}{2}(e_{\xi 1}{}^{T}e_{\xi 1}+e_{\xi 2}{}^{T}e_{\xi 2})$, an upper bound of Equation (38) is obtained as follows:

(40) $$\begin{array}{ll} \dot{V}(\Gamma_{1},t) & \leq -\kappa {\left\|\Gamma_{1}\right\|}^{2}+\left\|e_{\xi 3}\right\|\rho \left\|\Gamma_{1}\right\|-e_{\xi 3}{}^{T}K_{s1}e_{\xi 3}\\ & \leq -(\kappa -\frac{\rho^{2}}{4\vartheta }){\left\|\Gamma_{1}\right\|}^{2} \end{array}$$

The positive constants κ, ϑ are defined as $\kappa =\min\{1,\sigma_{i}-\frac{1}{2},\lambda_{i}-\frac{1}{2}\}$ and $\vartheta =\min\{K_{s1i}\}(i=1,2,3)$.

Therefore:

(41) $$\dot{V}(\Gamma_{1},t)\leq -l{\left\|\Gamma_{1}\right\|}^{2}\;\mathrm{for}\;\vartheta >\frac{\rho^{2}}{4\kappa },$$

where l is a positive constant. According to the Lyapunov method, the proposed outer loop control method is stable and the tracking error converges to zero.

### 2.3. Inner Loop Attitude Tracking Control

The description of the inner loop control system of the quadrotor is shown in Equation (9). Let the attitude tracking error $e_{\eta 1}$ and the filtered error signals $e_{\eta 2}$, $e_{\eta 3}$ be defined as follows [36,37]:

(42) $$e_{\eta 1}=\eta_{d}-\eta ,$$

(43) $$e_{\eta 2}={\dot{e}}_{\eta 1}+\gamma e_{\eta 1},$$

(44) $$e_{\eta 3}={\dot{e}}_{\eta 2}+\delta e_{\eta 2},$$

where ${\dot{e}}_{\eta 2}={\ddot{e}}_{\eta 1}+\gamma {\dot{e}}_{\eta 1}$, ${\ddot{e}}_{\eta 1}={\ddot{\eta }}_{d}-\ddot{\eta }$ and ${\dot{e}}_{\eta 1}={\dot{\eta }}_{d}-\dot{\eta }$, which is determined by the error of input and output data of the system. $\gamma =diag\{\gamma_{1},\gamma_{2},\gamma_{3}\}$, $\delta =diag\{\delta_{1},\delta_{2},\delta_{3}\}$ are positive definite diagonal matrices, and $\gamma_{i}>\frac{1}{2},\delta_{i}>\frac{1}{2},i=1,2,3$.

Combine the Equations (42)–(44) and (9) obtain Equation (45):

(45) $$J{\dot{e}}_{\eta 3}=J\delta {\dot{e}}_{\eta 2}+J\gamma {\ddot{e}}_{\eta 1}+J{\dddot{\eta }}_{d}+K_{\eta }\ddot{\eta }-\dot{\tau }-{\dot{d}}_{\eta }.$$

Define the auxiliary equations $N_{2}(t)$, $N_{d2}(t)$, ${\tilde{N}}_{2}(t)$ as Equations (46)–(48):

(46) $$N_{2}(t)=K_{\eta }\ddot{\eta }-{\dot{d}}_{\eta }+J{\dddot{\eta }}_{d}+J\gamma {\ddot{e}}_{\eta 1}+J\delta {\dot{e}}_{\eta 2}+e_{\eta 2},$$

(47) $$N_{d2}(t)=K_{\eta }{\ddot{\eta }}_{d}-{\dot{d}}_{\eta }+J{\dddot{\eta }}_{\;d},$$

(48) $${\tilde{N}}_{2}(t)=N_{2}(t)-N_{d2}(t)=-K_{\eta }{\ddot{e}}_{\eta 1}+J\gamma {\ddot{e}}_{\eta 1}+J\delta {\dot{e}}_{\eta 2}+e_{\eta 2}.$$

According to Equations (46)–(48), Equation (45) can be written as (49):

(49) $$J{\dot{e}}_{\eta 3}=-e_{\eta 2}-\dot{\tau }+N_{d2}(t)+{\tilde{N}}_{2}(t).$$

According to Remark 2, we know that $N_{d2}(t),{\dot{N}}_{d2}(t)\in L_{\infty }$, and $N_{2}(t)$ is continuously differentiable. According to the mean-value theorem in [36], so ${\tilde{N}}_{2}(t)$ is bounded as in Equation (50):

(50) $$\left\|{\tilde{N}}_{2}(t)\right\|\leq \rho_{2}\left\|\Gamma_{2}\right\|,$$

where $\left\|\cdot \right\|$ represents the Euclidean norm, $\Gamma_{2}={\left[e_{\eta 1},e_{\eta 2},e_{\eta 3}\right]}^{T}$ is the error vector, and $\rho_{2}$ is a positive constant.

The same as Section 2, the saturation function (25) is introduced to replace the sign function. The final RISE attitude controller is designed as follows:

(51) $$\tau =\int_{0}^{t}(K_{s2}+I_{3\times 3})e_{\eta 3}d\tau +\int_{0}^{t}\beta_{2}\tanh(e_{\eta 2})d\tau .$$

Equation (51) is only related to the system error data $e_{\eta 1}$ and its filtered error signals $e_{\eta 2}$, $e_{\eta 3}$ but does not rely on the system model. Therefore, the proposed inner loop attitude RISE algorithm is also a strictly data-driven model-free control method.

The stability proof is the same as in Section 2. According to the Lyapunov method, the proposed inner loop attitude control method is stable and the tracking error converges to zero.

## 3. Data-Driven Model-Free Adaptive Control Method of Quadrotor Formation Based on ISMC

In the Section 2, the method of single quadrotor trajectory tracking including outer loop position control and inner loop attitude control is introduced in detail. Therefore, formation control in this paper only concerns the control of position and velocity. According to literature [39,40], the UAV is regarded as a point-mass system at the formation control level in this paper, and the double integrator model is shown in Equation (52):

(52) $$\left\{\begin{array}{c} {\dot{P}}_{Fi}=v_{Fi}\\ {\dot{v}}_{Fi}=u_{Fi} \end{array}\right.,$$

where $P_{Fi}$, $v_{Fi}$, $u_{Fi}$ are the position, velocity, and control input of the followers.

**Figure 3:**
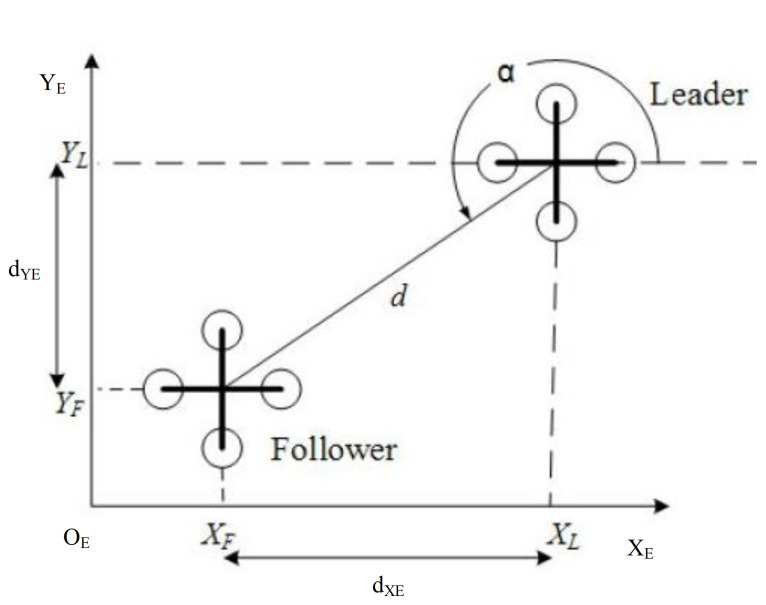
Schematic diagram of quadrotor formation distance.

Consider a formation n∈{L,1,2,3,4,⋯N} composed of n UAVs, using a leader-follower formation strategy, where L represents the leader and N represents the number of follower. The height in the Z direction can be the same or different when the followers track the leader. In the X-Y plane, the distance between the follower and the leader is d and the angle is α. Figure 3 is a schematic diagram of the quadrotor formation world coordinate system distance, $d_{XE},d_{YE}$ is the distance value of d projected on the $X_{E},Y_{E}$ axis of the world coordinate system, then the value of $d_{XE},d_{YE}$ in the body coordinate system $d_{XB},d_{YB}$ is shown in Equations (52) and (53):

(53) $$d_{XB}=-(X_{L}-X_{F})\cos(\psi_{L})-(Y_{L}-Y_{F})\sin(\psi_{L}),$$

(54) $$d_{YB}=(X_{L}-X_{F})\sin(\psi_{L})-(Y_{L}-Y_{F})\cos(\psi_{L}).$$

**Figure 4:**
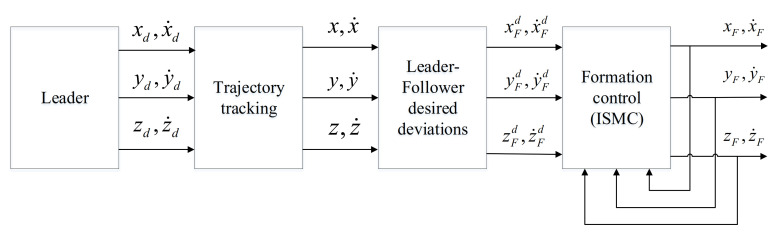
Formation control system structure.

The leader-follower performs formation control. Leader performs the trajectory tracking control in the Section 2. The followers obtain the desired position according to the information of the leader after trajectory tracking and the desired deviations from leader. The expected position is input to the formation controller to realize the cooperative control of the formation. The control system structure diagram is shown in Figure 4, and the formation control error satisfies the Equation (55):

(55) $$\begin{array}{l} \underset{t\to \infty }{\lim}\left\|e_{X}\right\|=\left\|d_{XB}^{d}-d_{XB}\right\|=0\\ \underset{t\to \infty }{\lim}\left\|e_{Y}\right\|=\left\|d_{YB}^{d}-d_{YB}\right\|=0\\ \underset{t\to \infty }{\lim}\left\|e_{Z}\right\|=\left\|d_{ZB}^{d}-d_{ZB}\right\|=0 \end{array}$$

where $d_{XB}^{d}$, $d_{YB}^{d}$, $d_{YB}^{d}$ are the expected distances of the body coordinate system X, Y, and Z. The SMC balance control is adopted for the system (55), and the design sliding surface is (56):

(56) $$s={\dot{e}}_{Fi}+\mu e_{Fi},$$

where s is the sliding surface, $e_{Fi}$ is the position error data of follower, μ is outer loop sliding mode surface control parameters.

From Equations (55) and (56), the exponential approach law (57) is used, and formation control disturbance $d_{F}$ is added, then the control quantity $u_{Fi}$ is obtained as shown in Equation (58):

(57) $$\dot{s}=-ks-\varepsilon sign(s),$$

where k is the exponential approach law parameter; ε is symbolic function parameter.

(58) $$u_{Fi}={\ddot{P}}_{L}+\mu {\dot{e}}_{Fi}+ks+(c+\varepsilon )sign(s)+d_{F},$$

where ${\ddot{P}}_{L}$ is 2th derivative of the position with respect to time after trajectory tracking of the leader; c is symbolic function adjustment parameter.

Equation (58) uses the system error data ${\dot{e}}_{Fi}$, but contains the symbolic function $\mathrm{sign}(s_{i})$ and the model-related quantity ${\ddot{P}}_{L}$, which still depends on the mathematical model of the UAV and has a chattering problem. Therefore, let ${P=\ddot{P}}_{L}+d_{F}$ adopt the adaptive update law (59) and saturation function (25) to obtain the ISMC algorithm as shown in Equation (60):

(59) $$\dot{\hat{P}}=s=({\dot{e}}_{Fi}+\mu e_{Fi}),$$

(60) $$u_{Fi}=\mu {\dot{e}}_{Fi}+ks+(c+\varepsilon )\tanh(s)+\hat{P}.$$

Equation (60) is only related to the system error data $e_{Fi}$, ${\dot{e}}_{Fi}$, and it does not depend on the system model. Therefore, the proposed quadrotor formation cooperative control ISMC algorithm is strictly a data-driven model-free control method.

To prove the stability of the proposed method, define the Lyapunov function:

(61) $$V_{1}=\frac{1}{2}s^{2}.$$

Differentiate it get:

(62) $${\dot{V}}_{1}=s\dot{s}.$$

From Equation (56):

(63) $${\dot{V}}_{1}=s\dot{s}=s(-ks-\varepsilon sign(s))=-ks^{2}-\varepsilon \left|s\right|<0.$$

Therefore, according to the Lyapunov method, the proposed UAV formation cooperative control method is stable and the tracking error converges to zero.

## 4. Simulation

A model-free adaptive trajectory tracking control method based on the RISE and ISMC algorithm using quadrotor formation data is adopted. With quadrotor as the control object, the leader performs trajectory tracking control, and the leader-follower performs formation collaborative control is verified by simulation experiment. In the simulation, the parameters of the quadrotor are taken as m=0.65 kg, $g=9.81\;m/s^{2}$, d=0.2 m, $k_{c}=3.1\times 10^{-7}\;Nms^{2}/rad^{2}$, $K_{\xi 1}=K_{\xi 2}=K_{\xi 3}=0.01\;Ns/m$, $K_{\eta 1}=K_{\eta 2}=K_{\eta 3}=0.1\;kgm^{2}/s$, $J_{x}=J_{y}=7.5\times 10^{-3}\;kgm^{2}$, $J_{z}=1.3\times 10^{-2}\;kgm^{2}$.

In the simulation, the yaw angle is fixed to zero during the entire trajectory tracking process, the reference trajectory $Y_{d}(t)={\left[x_{d}(t),y_{d}(t),z_{d}(t),\psi_{d}(t)\right]}^{T}$ of the leader is shown in Equation (64), the follower and the leader maintain the expected deviation, and the initial state of the followers is shown in Equations (65) and (66):

(64) $$Y_{d}(t)={\left[0,0,0.1t,0\right]}^{T},$$

(65) $$\begin{array}{l} d_{1}(t)={\left[6\sin(0.1t+\frac{\pi }{3}),6\cos(0.1t+\frac{\pi }{3}),0,0\right]}^{T}\\ d_{2}(t)={\left[6\sin(0.1t+\frac{2\pi }{3}),6\cos(0.1t+\frac{2\pi }{3}),0,0\right]}^{T}\\ d_{3}(t)={\left[6\sin(0.1t+\frac{3\pi }{3}),6\cos(0.1t+\frac{3\pi }{3}),0,0\right]}^{T}\\ d_{4}(t)={\left[6\sin(0.1t+\frac{4\pi }{3}),6\cos(0.1t+\frac{4\pi }{3}),0,0\right]}^{T}\\ d_{5}(t)={\left[6\sin(0.1t+\frac{5\pi }{3}),6\cos(0.1t+\frac{5\pi }{3}),0,0\right]}^{T}\\ d_{6}(t)={\left[6\sin(0.1t+\frac{6\pi }{3}),6\cos(0.1t+\frac{6\pi }{3}),0,0\right]}^{T} \end{array}$$

(66) $$\begin{array}{l} Y_{1,0}(t)={\left[6\sin(\frac{\pi }{3}),3,0,0\right]}^{T}\\ Y_{2,0}(t)={\left[6\sin(\frac{2\pi }{3}),-3,0,0\right]}^{T}\\ Y_{3,0}(t)={\left[0,-6,0,0\right]}^{T}\\ Y_{4,0}(t)={\left[6\sin(\frac{4\pi }{3}),-3,0,0\right]}^{T}\\ Y_{5,0}(t)={\left[6\sin(\frac{5\pi }{3}),3,0,0\right]}^{T}\\ Y_{6,0}(t)={\left[0,6,0,0\right]}^{T} \end{array}$$

Set the control parameters as follow: λ=diag{2,2,1.5}, σ=diag{1,1,1.5}, $K_{s1}=diag\{4,4,12\}$, $I_{3\times 3}=diag\{1,1,1\}$, $\beta_{1}=diag\{3,3,4\}$, γ=diag{50,50,15}, δ=diag{50,50,20}, $K_{s2}=diag\{10,5,10\}$, $\beta_{2}=diag\{4,5,10\}$, μ=diag{26,26,13}, k=diag{10,7,10}, c=diag{4,4,4}, ε=diag{1,1,1}, $r_{\phi }=1$ adding disturbance as shown in Equation (67):

(67) $$\left\{\begin{array}{c} d_{\xi }=2\;(N),t>50s;d_{\xi }=0\;(N),0<t\leq 50s\\ \begin{array}{l} d_{\eta }=0.6\sin(t+20)+0.6\cos(t+20)\;(Nm)\\ d_{F}=10\exp(-\frac{1}{2}\;\frac{{\left(t-10\right)}^{2}}{0.1^{2}})+\;10\exp(-\frac{1}{2}\;\frac{{\left(t-60\right)}^{2}}{0.1^{2}})\;(N) \end{array} \end{array}\right..$$

**Figure 5:**
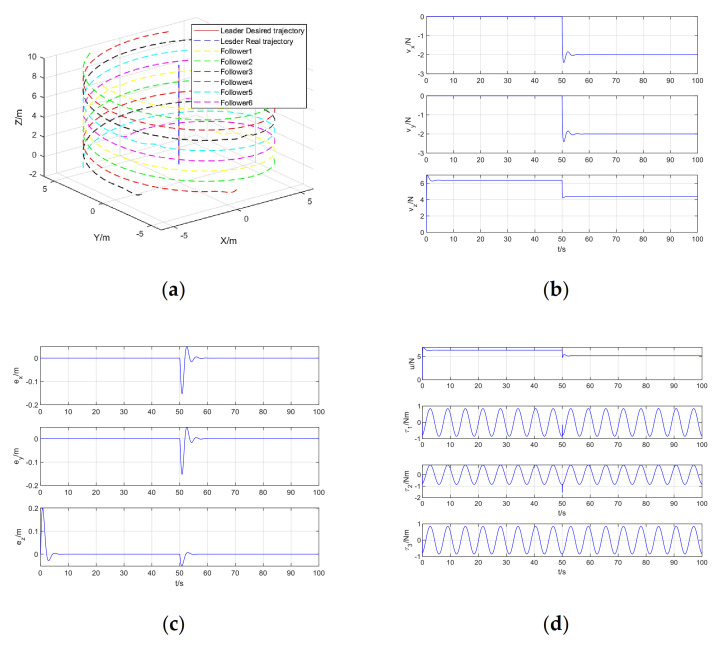
Simulation results of quadrotor formation cooperative trajectory tracking: (**a**) Formation cooperative trajectory tracking; (**b**) Virtual control input; (**c**) Trajectory tracking error; (**d**) Control input.

The simulation results of quadrotor formation cooperative trajectory tracking, including formation cooperative trajectory tracking, virtual control input, trajectory tracking error, and control input are shown in Figure 5a–d, respectively.

From Figure 5a, it can be seen that in the presence of position step disturbance and attitude periodic disturbance, the model-free adaptive control method of quadrotor trajectory tracking data-driven based on RISE algorithm perfectly realizes the trajectory tracking control of quadrotor. It can be seen from Figure 5b that the virtual control input changes rapidly at zero time and after the step disturbance is added to regulate the stability of the system. After the system is stable, the virtual control input is almost unchanged. From Figure 5c, it can be seen that the system error changes significantly at the zero time and after the step disturbance is added, and then the tracking error quickly converges to zero to achieve stable tracking. Figure 5d shows the actual control input, including total lift and rotational torque, which manifests as step changes and periodic changes. This is due to the step disturbance in the position and the periodic disturbance in the attitude Euler angle.

**Figure 6:**
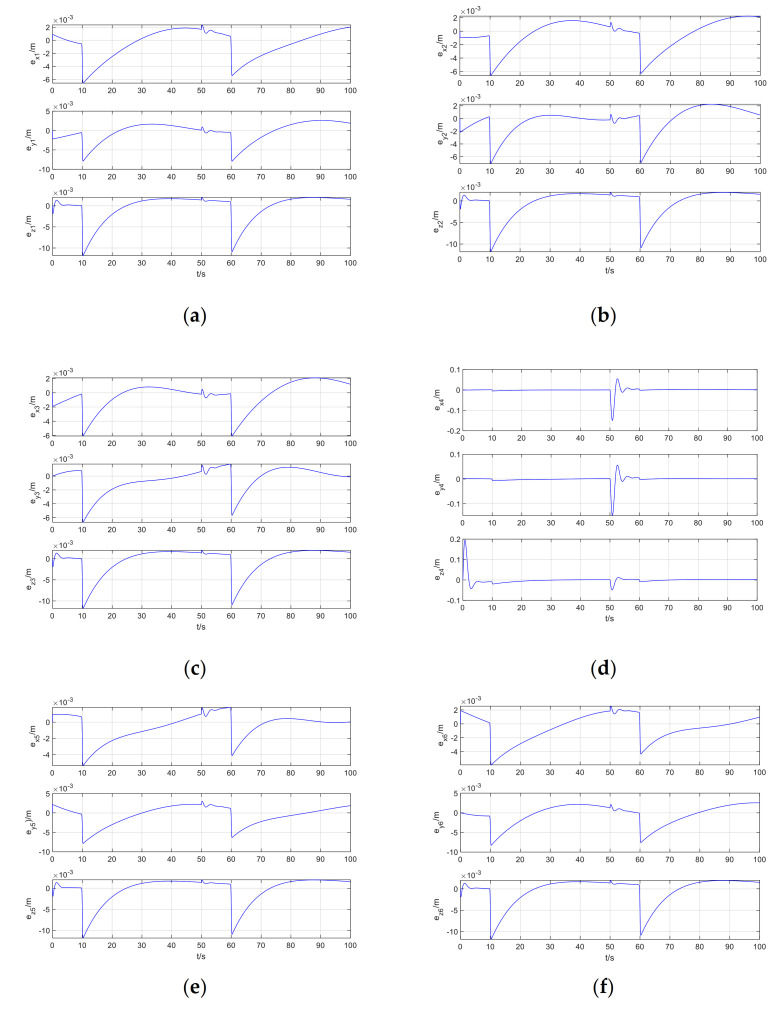
Follower1-Follower6 tracking error: (**a**) Follower1; (**b**) Follower2; (**c**) Follower3; (**d**) Follower4; (**e**) Follower5; (**f**) Follower6.

The tracking error of Follower1–Follower6 is shown in Figure 6.

It can be seen from Figure 5a that when the leader has position step disturbance, attitude periodic disturbance, and the followers has exponential decay disturbance, the leader and the follower maintain the expected position deviation, the data-driven model-free adaptive trajectory tracking control method based on RISE and ISMC perfectly realizes the coordinated trajectory tracking control of the quadrotor formation. It can be seen from Figure 6 that at zero time, after adding step disturbance time and exponential disturbance time, the position error of the followers suddenly converges to zero after a sudden change. From Figure 6d, it can be seen that due to different expected position errors of different followers, the position deviation changes can be quite different from other UAVs. However, careful observation reveals that the mutation point is still at zero moment, the step disturbance is added, and where the sudden change of exponential disturbance.

## 5. Discussion

**Figure 7:**
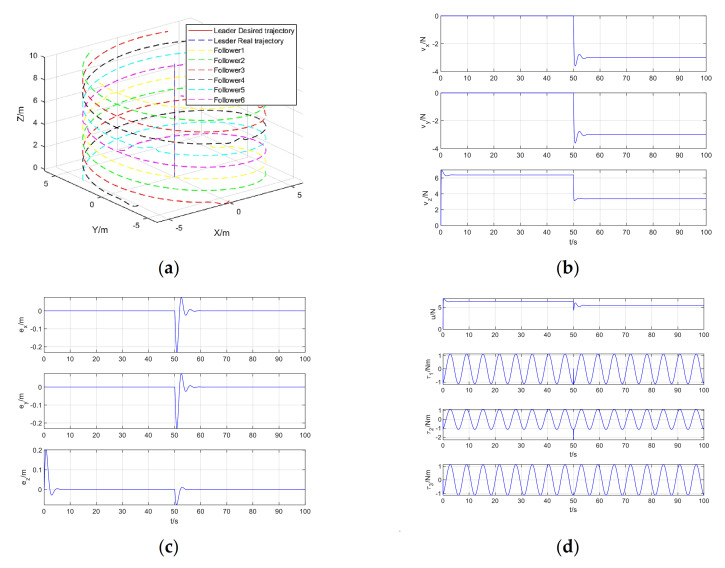
Simulation results of quadrotor formation cooperative trajectory tracking when increase the disturbance: (**a**) Formation cooperative trajectory tracking; (**b**) Virtual control input; (**c**) Trajectory tracking error; (**d**) Control input.

Increasing the disturbance in this paper is shown in Equation (68). The simulation results of quadrotor formation coordinated trajectory tracking, including formation coordinated trajectory tracking, virtual control input, trajectory tracking error, and control input are shown in Figure 7a–d:

(68) $$\left\{\begin{array}{c} d_{\xi }=3\;(N),t>50s;d_{\xi }=0\;(N),0<t\leq 50s\\ \begin{array}{l} d_{\eta }=0.8\sin(t+20)+0.8\cos(t+20)\;(Nm)\\ d_{F}=12\exp(-\frac{1}{2}\;\frac{{\left(t-10\right)}^{2}}{0.1^{2}})+\;12\exp(-\frac{1}{2}\;\frac{{\left(t-60\right)}^{2}}{0.1^{2}})\;(N) \end{array} \end{array}\right..$$

It can be seen from Figure 7 that when the position step disturbance and the attitude periodic disturbance are added, the virtual control input and the control input change accordingly to provide the amount of control required after the disturbance increases, and the trajectory tracking error does not change significantly. It can still achieve better trajectory tracking control.

**Figure 8:**
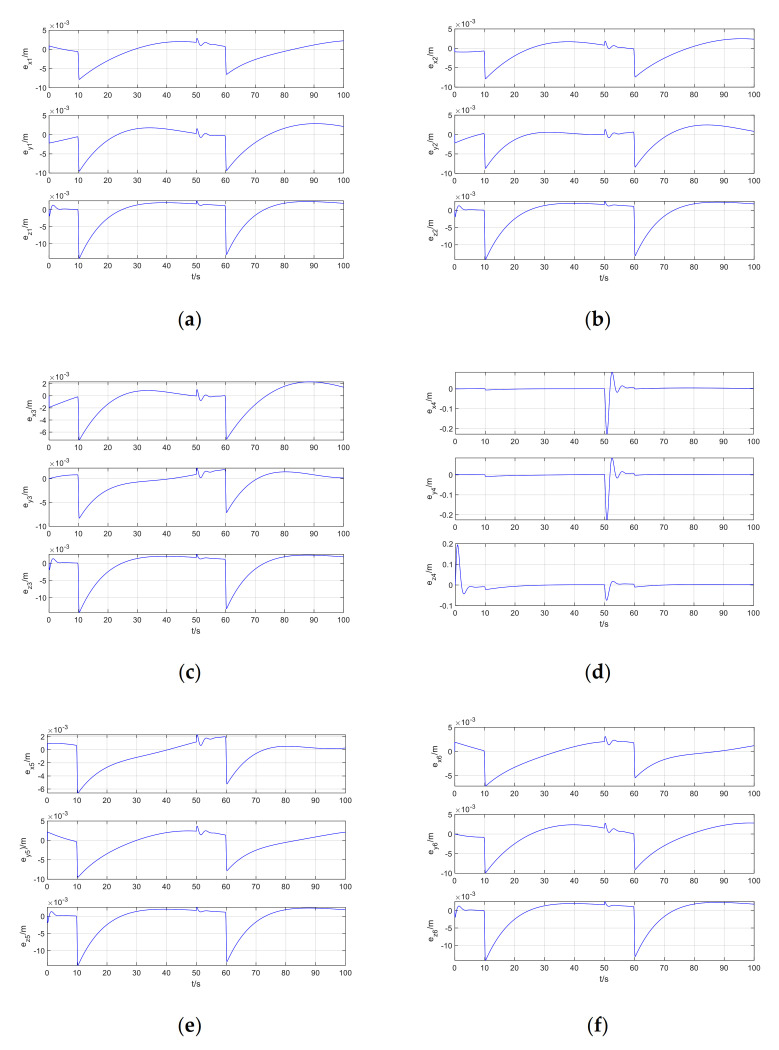
Follower1-Follower6 tracking error when increase the disturbance: (**a**) Follower1; (**b**) Follower2; (**c**) Follower3; (**d**) Follower4; (**e**) Follower5; (**f**) Follower6.

The tracking error of Follower1-Follower6 is shown in Figure 8.

It can be seen from Figure 8 that after increasing the disturbance of the formation cooperative control, the tracking error at the disturbance of the followers do not increase significantly, and it quickly converges to zero. It can also be seen from Figure 7a that the disturbance increases. The method proposed in this paper can still perfectly realize the coordinated trajectory tracking control of the formation, and the result of the coordinated formation trajectory tracking hardly changes. Therefore, the method proposed in this paper has good stability and robustness.

In addition, the RISE+RISE+ISMC method used in this paper is compared with the PD+PD+PD method. In the simulation, the parameters of the quadrotor remain unchanged, and the controller parameters are set to: PD+PD+PD trajectory tracking inner and outer loops and formation coordination control parameters are all set to $K_{p}=diag\{14,14,8\}$, $K_{d}=diag\{5,5,3\}$.

**Figure 9:**
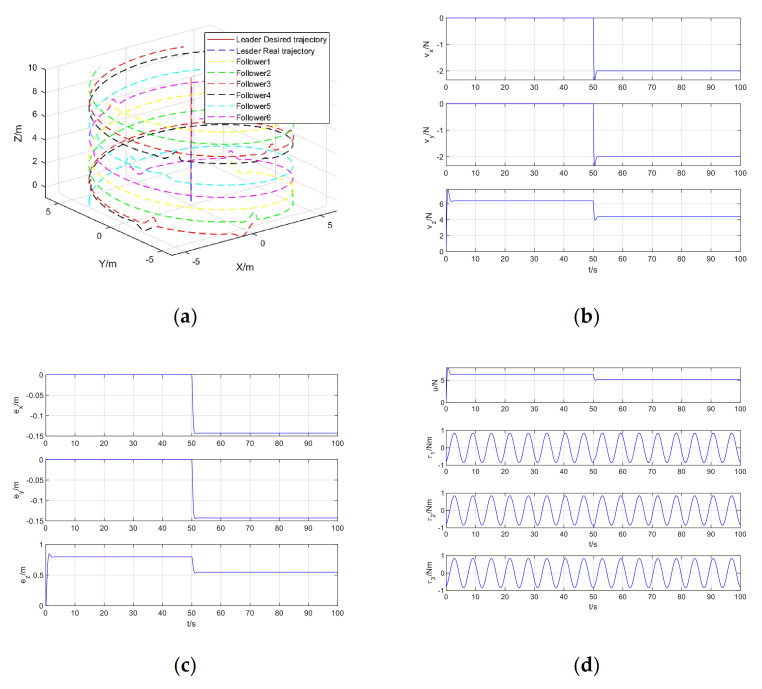
Simulation results of quadrotor formation cooperative trajectory tracking when use PD control: (**a**) Formation cooperative trajectory tracking; (**b**) Virtual control input; (**c**) Trajectory tracking error; (**d**) Control input.

The simulation results of quadrotor formation cooperative trajectory tracking, including formation cooperative trajectory tracking, virtual control input, trajectory tracking error and control input are shown in Figure 9a–d, respectively.

It can be seen from Figure 5 and Figure 9 that compared with the RISE method proposed in this paper, the virtual control input and control input provided by the general PD control cannot better offset the disturbance interference, the leader trajectory tracking steady-state error is not zero, the stability and robustness are poor, and the leader trajectory tracking control cannot be achieved well.

**Figure 10:**
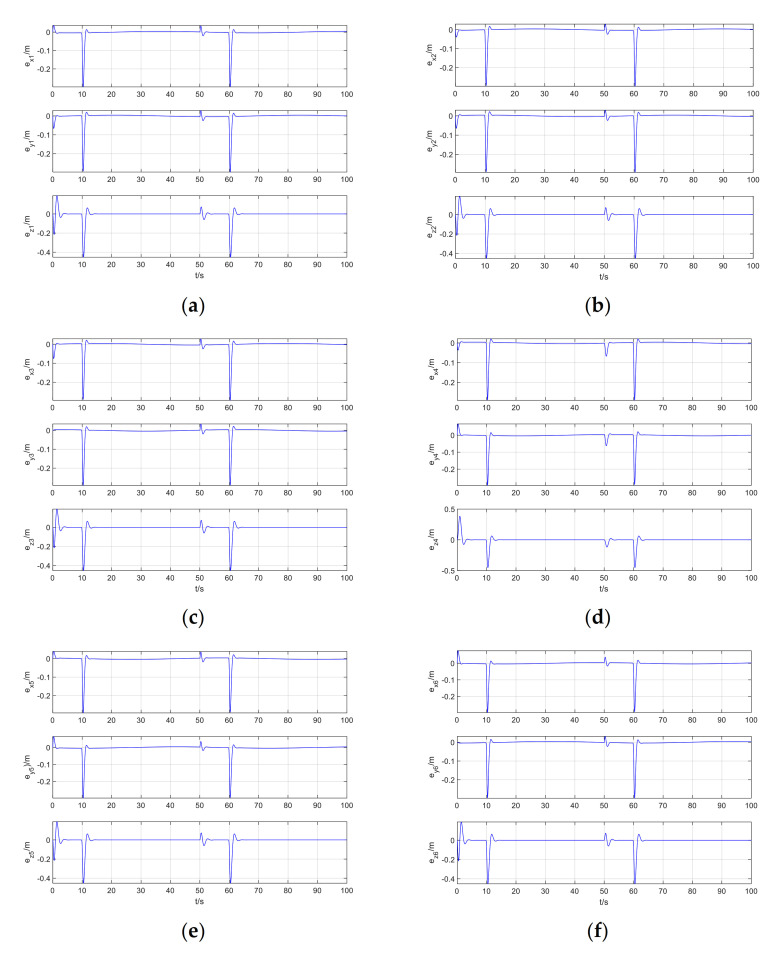
Follower1-Follower6 tracking error when use PD control: (**a**) Follower1; (**b**) Follower2; (**c**) Follower3; (**d**) Follower4; (**e**) Follower5; (**f**) Follower6.

The tracking error of Follower1-Follower6 is shown in Figure 10.

It can be seen from Figure 6 and Figure 10 that compared with the ISMC method proposed in this paper, the general PD control formation coordinated control steady-state error can still converge to zero, but the formation tracking error at the disturbance is significantly increased. Combined with Figure 9a, it can be seen that the general PD control can hardly realize the coordinated trajectory tracking control of the quadrotor formation.

## 6. Conclusions

This research proposes a data-driven model-free adaptive trajectory tracking control method for quadrotor formation based on RISE and ISMC to conduct collaborative trajectory tracking experiment and performance analysis for quadrotor formation. Using the leader-follower strategy, the leader performs trajectory tracking control, and the followers perform coordinated formation control. The trajectory tracking of the leader adopts the RISE method in the loop to eliminate interference and this method only uses the input and output data of the UAV system, and does not rely on any UAV dynamics kinematics model; the followers perform formation coordinated control and introduce an adaptive updated law and a saturation function to improve the SMC, eliminating the SMC algorithm controller design that depends on the mathematical model of the UAV and has chattering problems. The simulation results show that the designed quadrotor formation cooperative trajectory tracking control method is effective, and can effectively realize the tracking control of the quadrotor formation cooperative trajectory tracking. At the same time, the design of the controller does not depend on the quadrotor kinematics and dynamics models, it has high control accuracy and has broad prospects in practical applications. In addition, the data-driven method of quadrotor formation changes, formation obstacle avoidance, and formation parameters identification are also very interesting and challenging issues.

## Data Availability

Not applicable.
